# Case report: Mitral valve replacement for Libman-Sacks endocarditis and cerebral embolism of primary antiphospholipid syndrome

**DOI:** 10.3389/fcvm.2022.985111

**Published:** 2022-08-18

**Authors:** Huili Liang, Chunyan Ma, Xin Chen

**Affiliations:** Department of Cardiovascular Ultrasound, Clinical Medical Research Center of Imaging in Liaoning Province, The First Affiliated Hospital of China Medical University, Shenyang, China

**Keywords:** antiphospholipid syndrome, Libman-Sacks endocarditis, cerebral embolism, mitral valve, mitral regurgitation

## Abstract

Antiphospholipid syndrome (APS) is a systemic autoimmune disease characterized by recurrent arteriovenous thrombosis and/or morbid pregnancy. Valve involvement is the most common cardiac manifestation of APS, with lesions characterized by valve thickening and vegetations known as Libman-Sacks endocarditis (LSE). This report discussed a rare case of a 26-year-old young woman diagnosed with primary APS with multiple cerebral infarctions and right middle cerebral artery occlusion that occured 3 years ago. During the investigation, transthoracic echocardiography (TTE) revealed vegetations on both leaflets of the mitral valve with mild to moderate mitral regurgitation. One year following corticosteroid and anticoagulant treatment, mitral valve fibrosis and moderate to severe regurgitation were noted, after which mitral mechanical valve replacement was finally performed. Accordingly, this report suggests that LSE occurrence should be alerted during the examination of APS patients especially in those with cerebrovascular disease. Furthermore, establishing an early diagnosis and conducting close follow-ups are necessary for its timely intervention and treatment.

## Introduction

Libman-Sacks endocarditis (LSE) is a common manifestation in patients with antiphospholipid syndrome (APS) who have cardiac valve involvement; however, it is often ignored due to its asymptomatic nature during the early stages. As the disease progresses, serious complications such as valve dysfunction and cerebrovascular embolism, may occur (1). Therefore, achieving an early and accurate LSE diagnosis as well as performing timely treatment in patients with APS are crucial in preventing disease progression and improving prognosis (2). This report discusses a rare case of LSE complicated by cerebral embolism in a patient with primary APS who underwent mechanical mitral valve replacement.

## Case presentation

A 26-year-old young woman was hospitalized in the Rheumatology and Immunology department of a local hospital due to elevated blood pressure, elevated creatinine and head and neck erythema 5 years ago (Table 1). Laboratory tests revealed that the rheumatism antibody test was negative, and anti-β2 glycoprotein-1 antibody (anti-β2GP1) was >90 Umol/L. Because the patient had no history of abortion or oral estrogen-containing hormonal contraception and lacked diagnostic criteria, such as recurrent arteriovenous thrombosis, probable APS was diagnosed. The patient was then treated with 10 mg/days oral methylprednisolone. Two years later, the patient suffered from sudden slurred speech, numbness, and motor dysfunction in her right hand and numbness in her left lower extremities 2 months ago. Then, she was subsequently admitted to our hospital. An emergency brain computed tomography (CT) demonstrated multiple lacunar cerebral infarctions. Moreover, her physical examination demonstrated red spots on the head and neck with associated pruritus. Electrocardiography (ECG) showed sinus rhythm with 87 bpm. Her laboratory investigations revealed the following biochemical indicators: anti-β2 glycoprotein-1 antibody (anti-β2GP1)-IgG 210.5 CU (0.0–20.0 CU); anticardiolipin antibody (aCL)-IgG, aCL-IgA were increased with aCL-IgG 468.9 CU (0.0–20.0 CU), aCL-IgM 4.3 CU (0.0–20.0 CU), aCL-IgA 24.4 CU (0.0–20.0 CU) and positive lupus anticoagulant (LA). Prothrombin time was 37.4 s (11.0–14.3 s), and activated partial thromboplastin time was increased with 54.3 s (32.0–43.0 s). Creatinine was 133 μmol/L (41–73 μmol/L). However, testing for antinuclear antibody, anti-double-stranded DNA antibody, anti-U1RNP antibody, anti-SSA antibody, and anti-SSB antibody yielded negative results. The levels of hemoglobin, platelets, white cell count, C-reactive protein (CRP), erythrocyte sedimentation rate (ESR), C3 and C4, protein C and S, and urea and liver function indicators were within normal limits.

**TABLE 1:** Timeline of the patient’s clinical course.

2017	Diagnosed with probable APS at a local hospital. Oral methylprednisolone was administered
December 2019	Sudden slurred speech, numbness and motor dysfunction in the right hand and numbness in the left lower extremities 2 months ago
3 December 2019	Emergency brain CT scan showed multiple lacunar cerebral infarctions. The patient was then hospitalized
5 December 2019	MRI showed multiple infarctions and softening lesions in the right thalamus, paraventricular area, and cerebellar hemisphere. MRA showed right middle cerebral artery occlusion. Autoimmune indicators were suggestive for primary APS
10 December 2019	TTE showed mitral valve vegetations and mild to moderate mitral regurgitation
18 December 2019	The patient was discharged home, receiving prednisolone acetate and oral warfarin
July 2020	Chest tightness and shortness of breath were reported for 2 months. The patient was then rehospitalized
22 July 2020	Follow-up TTE showed moderate to severe mitral regurgitation
30 July 2020	Mitral valve mechanical valve replacement was performed
December 2021	Postoperative TTE revealed no mitral regurgitation with normal ventricular function

APS, antiphospholipid syndrome; CT, computed tomography; MRA, magnetic resonance arteriography; MRI, magnetic resonance imaging; TTE, transthoracic echocardiography.

**FIGURE 1:**
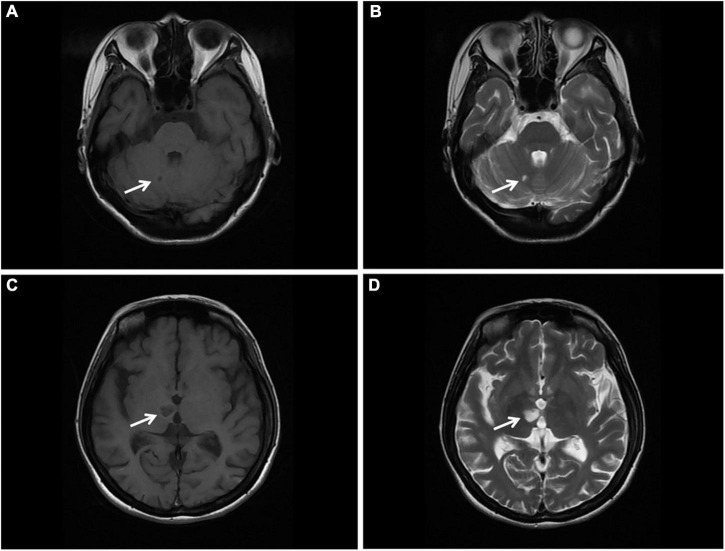
Brain MRI. **(A,B)** T1 and T2 sequences showing cerebral infarction with a clear border in the right cerebellar hemisphere (arrow). **(C,D)** T1 and T2 sequences showing cerebral infarction with a clear border in the right thalamus. MRI, magnetic resonance imaging (arrow).

**FIGURE 2:**
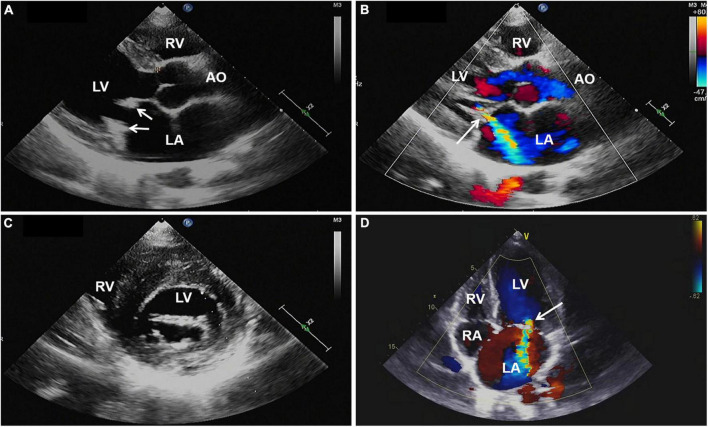
**(A,C)** Transthoracic echocardiography showing thickened mitral valve leaflets with verrucous, nodular, heterogeneous echoic vegetations located on the commissural border of both leaflet tips (arrows). **(B,D)** Color Doppler imaging revealing mild to moderate mitral regurgitation (arrow). AO, aorta; LA, left atrium; LV, left ventricle; RA, right atrium; RV, right ventricle; TTE, transthoracic echocardiography.

Magnetic resonance imaging (MRI) of the brain was then performed, showing multiple infarctions and softening lesions in the right thalamus, paraventricular area, and cerebellar hemisphere (Figure 1). Magnetic resonance arteriography (MRA) showed right middle cerebral artery occlusion. A carotid ultrasound scan was normal. Transthoracic echocardiogram (TTE) demonstrated that the cusps of the anterior and posterior mitral valve leaflets were thickened. Additionally, verrucous and nodular vegetations with heterogeneous echo-density could be seen at the commissural border of both mitral valve leaflets (Figures 2A,C). The vegetations were firmly attached to the surface of the valve without obvious independent motion. Mild to moderate mitral regurgitation was detected at the central commissure when the valve closed (Figures 2B,D) with an effective regurgitant orifice area (EROA) of 0.25 cm^2^ and the regurgitant volume of 36 mL. The other valves were morphologically normal. The left ventricular function was normal, with a left ventricular ejection fraction (LVEF) of 60%. Repeated blood cultures were negative, and the patient had no recent history of fever. Considering these findings, the patient was diagnosed with primary APS, LSE, and cerebral infarction. Symptomatic treatment was provided to her during hospital admission with sufficient low molecular weight heparin and warfarin anticoagulation. Oral prednisolone acetate (15 mg/day) and warfarin were administered after her discharge.

**FIGURE 3:**
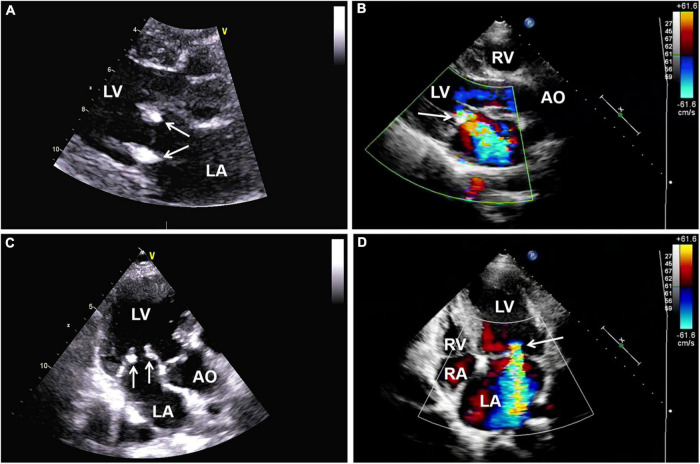
**(A,C)** Follow-up transthoracic echocardiography (TTE) demonstrating thickening and hyperechoic lesions of mitral valve leaflets (arrows), suggesting valvular fibrosis. **(B,D)** Color Doppler imaging showing moderate to severe regurgitation of the mitral valve. AO, aorta; LA, left atrium; LV, left ventricle; RA, right atrium; RV, right ventricle; TTE, transthoracic echocardiography.

**FIGURE 4:**
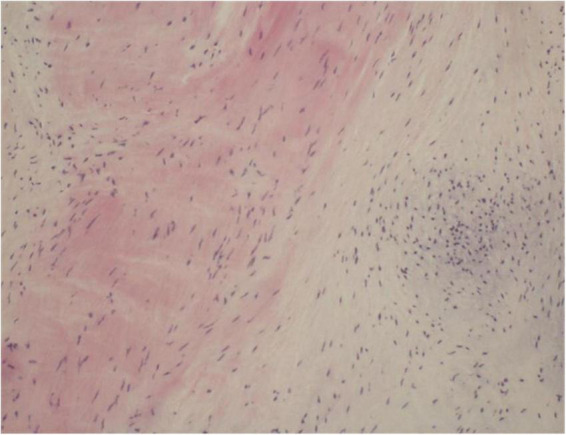
Histopathology of excised valve demonstrating fibrous tissue hyperplasia with hyaline degeneration and no inflammatory cell infiltration.

One year later, the patient again sought medical care due to chest tightness and shortness of breath for 2 months. Chest X-ray and chest CT were normal. Additionally, cardiac auscultation revealed a grade 3/6 apical systolic murmur. Follow-up TTE showed thickening and fibrosis of the anterior and posterior mitral valve cusps, mild stenosis (transmitral mean gradient of 3 mmHg and mitral valve area of 2.6 cm^2^) and moderate to severe regurgitation of the mitral valve (Figure 3) with EROA of 0.40 cm^2^ and the regurgitant volume of 58 mL. The other valves were normal. The left ventricular function was normal, with a left ventricular ejection fraction (LVEF) of 64%. Repeated blood cultures were again negative. Mitral valve mechanical valve replacement was then performed, which intraoperatively revealed thickening and multiple small nodular vegetations on the mitral valve. No perforation or destruction of the mitral valve was identified. Histopathology demonstrated fibrous tissue hyperplasia with hyaline degeneration and no inflammatory cell infiltration (Figure 4). Postoperatively, oral prednisolone acetate and warfarin were administered with an international normalized ratio (INR) target of 3.0–4.0. During her 17-month follow-up, the patient was clinically stable, the symptoms of cerebral infarction were relieved, and no new infarct was found on the follow-up brain CT. TTE revealed no mitral regurgitation with normal ventricular function.

## Discussion

Antiphospholipid syndrome is a non-inflammatory systemic autoimmune disease with an incidence of five new cases/100,000 persons per year and a prevalence of 40–50 cases/100,000 persons (3) and is more common in young women (4). “Definite” APS must have recurrent arteriovenous thrombosis and/or morbid pregnancy (habitual abortion, or stillbirth in the middle and late stages) as its main clinical manifestations (5). Additionally, laboratory tests should demonstrate persistently high titers of antiphospholipid antibodies, including aCL, LA, and anti-β2GP1 (5). APS can be divided into primary and secondary APS. Primary APS has been generally defined to cover diseases meeting the diagnostic criteria of APS without those that induce the production of antiphospholipid antibodies, such as autoimmune diseases and malignancies (5). APS occurring secondary to other diseases, such as systemic lupus erythematosus (SLE) and Sjogren syndrome, is traditionally termed secondary APS (5). Additionally, APS can also be divided into the following subtypes, including probable APS or pre-APS, seronegative APS, and catastrophic APS (6). The patient discussed in this case report was diagnosed with probable APS in a local hospital 5 years ago and was positive for antiphospholipid antibodies; however, there was a lack of certain components in the diagnostic criteria, such as thrombosis or recurrent abortion (6). While she was hospitalized 3 years ago, in conjunction with the results of her MRI, MRA and biochemical tests, she was diagnosed with primary APS. The clinical manifestations of APS are complex and diverse, and various body systems may be involved during the disease.

Involvement of the heart valve is the most common cardiac manifestation of APS, with 32% of APS cases demonstrating invasion of the heart valve and valvular damage (7). The lesions characterized by valve thickening and vegetations are defined as LSE. LSE is non-bacterial thrombotic endocarditis, which is most commonly seen in SLE, and a few cases have been reported in APS in recent years (2). Its pathogenesis involves the deposition of autoimmune complexes and complement, leading to valve thickening and the formation of fibrin-platelet thrombus on the valve. Antiphospholipid antibodies could activate endothelial cells, resulting in aggregation of monocytes and platelets, promoting the thrombosis of valves damaged by immune complex deposition, and aggravating valve damage and inflammatory changes (8). Echocardiography is the preferred investigation for LSE. Typical manifestations are verrucous and nodular vegetations of various sizes and shapes, which are usually small in size (<1 cm in diameter), and have irregular borders, heterogeneous echogenicity, and wide bases while being firmly attached to the valve surface (9). These are often located at the upstream, the commissural border of the valve leaflets, and can also involve the chordae tendineae and atrioventricular endocardial surface, without obvious voluntary motion (9). The mitral valve is most commonly involved, accounting for about 63% of cases, followed by the aortic valve, the tricuspid valve, and the pulmonary valve, which are rarely involved (10). Most patients have no obvious valve dysfunction with only mild regurgitation or less, and only 4–6% of patients have severe valve regurgitation (11). Zuily et al. have discovered that the incidence of LSE in the antiphospholipid antibody-positive group was three times that of the negative group, where moderate to severe mitral regurgitation was more likely to occur (12). Charles et al. have shown that the prevalence of LSE in double-positive or triple-positive patients is significantly higher than that in single-positive patients with antiphospholipid antibodies (13). All three antiphospholipid antibodies were positive in our patient, which might relate to the severity of valve regurgitation.

Studies have also found that LSE might be a common and under-recognized pathological process of embolic cerebrovascular disease, a potential embolic source of cerebrovascular embolism in patients with SLE and APS, and an independent risk factor of cerebrovascular events (8, 14, 15). Cerebrovascular events in patients with APS may be caused by the fragmentation of LSE or may be related to the hypercoagulable state caused by antiphospholipid antibodies (8). Erdozain et al. have shown that cerebrovascular events are more prevalent in patients with significant valvular lesions (15). In the present case report, the MRI scan exhibited small, multiple and scattered cerebral infarctions, while the MRA showed occlusion of the right middle cerebral artery. The cerebral vascular event of this patient was considered to be attributed to the combination of arterial embolism due to hypercoagulability and cardiogenic embolism as a result of LSE.

In patients with APS complicated by cerebrovascular embolism, early diagnosis and timely treatment of LSE are crucial. Controversy regarding LSE treatment in APS patients continues to exist. LSE treatment is usually performed using hormones, such as corticosteroids; however, studies have shown that corticosteroids can accelerate valve vegetation healing, which might lead to valve scarring and fibrosis, thus worsening valve dysfunction (4, 16). Therefore, scholars have suggested that corticosteroid therapy should not be recommended for APS valve lesions (4). Thus, for patients suffering from LSE with severe valvular regurgitation and cerebrovascular embolism, surgical treatment is still required to prevent further deterioration of the cardiac structure and function and recurrent embolism occurrence. Mechanical valve replacement continues to remain the preferred surgical treatment for young patients with LSE, and studies have shown that better outcomes are evident when performing mitral valve replacement compared to mitral valve repair (16). Native valve repair does not seem to alter the progression of valve thickening and calcification; hence, replacement is ultimately necessary. Regarding the patient in this case report, due to her poor response to medical treatment, the degree of mitral regurgitation was aggravated, which was further complicated by cerebral vascular embolism. Mitral valve mechanical valve replacement was performed to prevent the deterioration of the condition and recurrence of embolism. Due to the hypercoagulable state of APS, anticoagulants should be prescribed for long-term use following surgery to prevent thrombosis, with regular dosage readjustment.

## Conclusion

The findings of this case report suggest that clinicians should be more aware of LSE when performing echocardiography in patients with APS. Once the diagnosis is confirmed, close follow-up and timely treatment are necessary. Additionally, there were concerns that the use of corticosteroid agents may cause shrinking and scarring of heart valves; therefore, despite the indications for surgery for patients with LSE have not been sufficiently studied, severe valvular dysfunction, large vegetations and recurrent embolization despite therapeutic anticoagulation are generally accepted indications for surgical intervention.

## Data availability statement

The original contributions presented in this study are included in the article/supplementary material, further inquiries can be directed to the corresponding author.

## Ethics statement

Written informed consent was obtained from the individual(s) for the publication of any potentially identifiable images or data included in this article.

## Author contributions

HL: conceptualization, data interpretation, and drafting manuscript. XC: conceptualization, project administration, and supervision. CM: supervision. All authors have read and approved the submitted version.
